# APIS: The NIAAA Alcohol Policy Information System

**DOI:** 10.35946/arcr.v35.2.08

**Published:** 2014

**Authors:** Michael Hilton

**Affiliations:** **Michael Hilton, Ph.D.***is the deputy director of the Division of Epidemiology & Prevention Research, National Institute on Alcohol Abuse and Alcoholism, Bethesda, Maryland.*

The Alcohol Policy Information System (APIS) is an NIAAA-sponsored Web site that provides detailed information on alcohol-related public policies at both the State and Federal levels. Updated annually, the APIS information can be used to identify policy changes in 33 policy areas. Up to two-thirds of these policies can be tracked back to 1998, and data on the remaining one-third are available since 2003.

APIS provides a tool for conducting research on the effectiveness of alcohol policies in reducing alcohol-related harm. Natural experiments on the impact of a policy are possible whenever a State makes a change in some aspect of alcohol policy. To conduct such an experiment, scientists obtain a series of regularly collected data on an outcome of interest, compare problem levels before and after a policy change, and report this comparison for both the “experimental” State and a “control” State that did not change its policy. As described in other articles in this issue, measures of policy outcomes can be taken from a number of existing data series that report rates of mortality, morbidity, accidents, crimes, and the like. APIS provides an accurate and consistently maintained source of the “independent variable” of interest in these studies, showing changes in codified statutes and regulations.

As the lead Federal agency responsible for research into alcohol use and its related consequences, NIAAA believes that providing this research tool is critically important. A vigorous program of research on the effects of various drinking-and-driving policies has been a key factor in reducing alcohol-related traffic fatalities to drivers, passengers, and pedestrians, ages 16 to 20 by 50 percent since 1982. By providing information on other aspects of alcohol policy, APIS can lead to similar advances in research knowledge and improvements in public health.

APIS development began in September of 2001, and the Web site went public in June of 2003. The 33 existing policy topics cover underage drinking, blood alcohol concentration limits, transportation, taxation, retail sales, alcohol control systems, pregnancy and alcohol, and health care services and financing.

New topics are added periodically and are selected on the basis of the likelihood that they will stimulate new research on policy outcomes. Candidate policy topics should cover areas where recent policymaking activity has occurred or where current discussions imply that impending change is likely. Additional criteria include (1) a scientifically grounded expectation that policy change in the particular candidate area is likely to produce measurable changes in some public health outcome; (2) sufficient data are available to measure these policy outcomes; (3) the legal research involved in locating relevant statutes and regulations and in constructing precise and consistent operational definitions that categorize meaningful features of the laws is a manageable task that falls within the resources available; and (4) case law or administrative decision making that is not captured in statutory or regulatory texts plays a limited role in interpreting the policy.

Documenting the decision rules for categorizing the features of State policies is a critical concern. This documentation must capture the decisions made by the APIS research staff in such a way that consistent decisions can be made across all States, consistent decisions can be made across time as policies change, and the basis for the decisions can be clearly communicated to research users.

In addition to strict documentation, a hallmark of APIS has been careful attention to quality control. Each new law or regulation is reviewed by a minimum of two research lawyers and a senior social scientist. Any conflicting interpretations are then resolved. On occasion, new developments in some detail of a policy reveal a gap or an ambiguity in the coding procedures that were used previously. In suchcases, that coding is reviewed and, if necessary, changes are made. Likewise, when errors are encountered in APIS’s legal descriptions, those corrections are made and documented in a “Change Log.” Fortunately, the number of such instances has remained remarkably low.

Policy changes are reviewed each year for each topic and for every State. Shortly after the beginning of each calendar year, legal tools become available that make it possible to identify all of the codified statutory and regulatory activity that has occurred in a given State over the preceding year. APIS legal researchers sift through these policies to see if changes have occurred in any of the 32 policy areas tracked by APIS. Typically, the most difficult aspect of this work is “demonstrating the negative,” that is, verifying that there has not been a change in some specific aspect of a policy. This work is costly and has become the largest part of the APIS contract budget.

The APIS Web site (www.alcoholpolicy.niaaa.nih.gov) features three basic displays (see figure):

- “Data on a Specific Date” shows the features of a given policy as of the date of the last system-wide update (the default value) or as of a specific date selected by the viewer. Any given APIS policy will vary across States in a number of specific features. Understanding these details is important.
- “Changes Over Time” contains entries whenever a State changes its policy. There is one row showing the policy before the change and another showing the policy afterward. Should a State make additional policy changes, even more rows are added.
- “Timeline of Policy Changes” displays calendar years on the horizontal axis and States on the vertical axis. Icons indicate the year (and quarter) when a policy change went into effect. Note that for all APIS information, policies are categorized according to the date when they took effect rather than when they were enacted.

There are a number of other useful Web site features and displays. The “Maps and Charts” feature shows graphic depictions of selected information. An “About This Policy” section gives useful background information on each policy. “State Profiles of Underage Drinking Laws” contains brief State-by-State summaries of policy in 12 topic areas specifically related to underage drinking. “Citations” are given so that the statutory or regulatory text of each policy covered by the Web site canbe located. Finally, there are links to NIAAA’s current funding opportunity announcements, inviting researchers to submit proposals to study policy impacts. NIAAA encourages all researchers who are interested in the effect of public policy on alcohol-related health outcomes to visit the APIS Web site and to consider applying for research support.

## Figures and Tables

**Figure:**
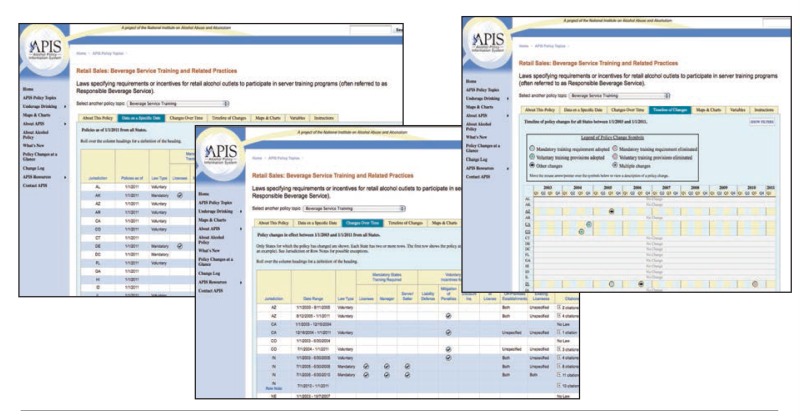
The APIS Web site (www.alcoholpolicy.niaaa.nih.gov) features three basic displays

